# Diversity of Extremely Halophilic Archaeal and Bacterial Communities from Commercial Salts

**DOI:** 10.3389/fmicb.2017.00799

**Published:** 2017-05-10

**Authors:** Ashagrie Gibtan, Kyounghee Park, Mingyeong Woo, Jung-Kue Shin, Dong-Woo Lee, Jae Hak Sohn, Minjung Song, Seong Woon Roh, Sang-Jae Lee, Han-Seung Lee

**Affiliations:** ^1^Major in Food Biotechnology, Division of Bioindustry, Silla UniversityBusan, South Korea; ^2^Department of Korean Cusine, Jeonju UniversityJeonju, South Korea; ^3^School of Applied Biosciences, Kyungpook National UniversityDaegu, South Korea; ^4^Research Center for Extremophiles and Marine Microbiology, Silla UniversityBusan, South Korea; ^5^Microbiology and Functionality Research Group, World Institute of KimchiGwangju, South Korea

**Keywords:** commercial salts, halophilic archaea, halophilic bacteria, Ethiopia, Korea, diversity

## Abstract

Salting is one of the oldest food preservation techniques. However, salt is also the source of living halophilic microorganisms that may affect human health. In order to determine the microbial communities of commercial salts, an investigation were done using amplicon sequencing approach in four commercial salts: Ethiopian Afdera salt (EAS), Ethiopian rock salt (ERS), Korean Jangpan salt (KJS), and Korean Topan salt (KTS). Using domain-specific primers, a region of the 16S rRNA gene was amplified and sequenced using a Roche 454 instrument. The results indicated that these microbial communities contained 48.22–61.4% Bacteria, 37.72–51.26% Archaea, 0.51–0.86% Eukarya, and 0.005–0.009% unclassified reads. Among bacteria, the communities in these salts were dominated by the phyla *Proteobacteria, Bacteroidetes, Actinobacteria*, and *Firmicutes*. Of the archaea, 91.58% belonged to the class *Halobacteria*, whereas the remaining 7.58, 0.83, and 0.01% were *Nanoarchaea, Methanobacteria*, and *Thermococci*, respectively. This comparison of microbial diversity in salts from two countries showed the presence of many archaeal and bacterial genera that occurred in salt samples from one country but not the other. The bacterial genera *Enterobacter* and *Halovibrio* were found only in Korean and Ethiopian salts, respectively. This study indicated the occurrence and diversity of halophilic bacteria and archaea in commercial salts that could be important in the gastrointestinal tract after ingestion.

## Introduction

Salting is one of the oldest food preservation methods, which is usually regarded as a technique to control microbial activity. However, some studies revealed that salt itself contains a wide range of halophilic organisms, including bacteria and archaea that are unique to these environments (Henriet et al., 2014) as well as salted and fermented foods (Park et al., 2009; Roh et al., 2010). In recent years, novel halophilic archaea namely *Halopiger thermotololerans* (Minegishi et al., 2016), *Haloparvum alkalitolerans* (Kondo et al., 2016), and *Halarchaeum grantii* (Shimane et al., 2015) were isolated from commercial salts using culture-based methods. Indeed, a variety of halophilic archaea were found in high salt-fermented foods and human intestines, suggesting that halophiles play an important role in food processing and preparation (Lee, 2013). Analogously, high-throughput microbial community studies demonstrated that diverse bacteria and archaea were also found in hypersaline or saline environments such as ancient halite (Schubert et al., 2010), estuarine sediments (Webster et al., 2015), lacustrine sediments (Gugliandolo et al., 2015), saline lakes (Li et al., 2015), saline soil (Walsh et al., 2005), seawater (Bougouffa et al., 2013), and solar salterns (Mutlu and Guven, 2015). In addition, there were isolation of archaea from primary salt crystal (Vreeland et al., 2007), middle late Eocene rock salt (Jaakkola et al., 2014), ancient halite (Schubert et al., 2010), and bacteria from primary salt crystal (Vreeland et al., 2000; Satterfield et al., 2005). In light of this, ecological and microbiological studies of commercial salts are of special interest because these can provide insights into the correlative microbial dynamics between hypersaline environments and salted and fermented foods. However, the whole microbiota of commercial salts still remains unclear.

Korean traditional foods like kimchi, seasoning pastes, soy sauce, and salted seafoods are seasoned exclusively with Shinan solar salt to maintain their taste and flavor. Some physicochemical and sensory characteristics of Korean fermented foods salted with domestic solar salts are distinct from those with imported salts. This might be a result of the specific microbiota in various salts as well as mineral concentration, although this speculation requires further investigation. The Danakil depression is one of the most ancient salt-producing areas in the world, and it is still the source of salt for more than 100 million people in Africa, especially in Ethiopia, Eritrea, and Djibouti. In addition, the area represents an unexplored hypersaline ecosystem that is unique in its geology, climate, and landscape features.

To our knowledge, there is no study on the occurrence and diversity of microbial communities in commercial salts from these two countries. These reasons tempted us to investigate and compare the composition of the microbial community and diversity in salts. To study the composition of microbial community and its diversity, a culture-independent amplicon sequencing approach seems to be more sensitive and effective than a conventional culture-dependent method (Streit and Schmitz, 2004; Bik, 2014). Several metagenomic studies revealed a fair number of microbial communities that were not found in a culture-based approach by the next-generation sequencing with the Roche/454 GSFLX Titanium platform (Streit and Schmitz, 2004; Jung and Kang, 2014; Suh et al., 2015). Such metagenomic studies can provide a comprehensive understanding of microbial survival and adaptation strategies in extreme environments and special ecosystems (Fierer et al., 2012; Neelakanta and Sultana, 2013). Here, we chose four kinds of commercial salts from two different areas in Korea (Topan and Jangpan) and Ethiopia (Afdera Lake and rock salt) to compare their microbial communities using a culture-independent approach and discuss the microbial diversity and community structure in salts from these regions.

## Materials and methods

### Site description and sample collection

Four commercially available salts, namely, Afdera, rock, Topan, and Jangpan samples, were collected directly from the companies that produce and distribute the salts in Ethiopia (Afar Salt and Bashenfer Salt) and Korea (Sumdleche Co. Korea). They were stored at room temperature in a dark and dry place until they were used in experiments. The Afdera and rock salts were from Ethiopia, and Topan and Jangpan were from Korea. The salts were designated based on the production process, source, and country of origin: Ethiopia Afdera Salt (EAS), Ethiopian Rock Salt (ERS), Korean Topan Salt (KTS), and Korean Jangpan Salt (KJS). The Ethiopian salts were produced in the Danakil depression, and the Korean salts were produced in Shinan, Korea. Shinan is located in the southeast of Korea at latitude 35° 12″ 30′ north and longitude 126° 23″ 00′ east, and has a length from east to west of 79.3 km and south to north of 65.6 km. The average temperature in January is 2–4°C and in August is 24°C. The average annual temperature is 14°C, and average annual rainfall is 800–1,000 mm. Two types of sun-dried salts, KTS and KJS are being produced in Shinan area. KTS is dried on the hardened mud-flat and KJS on the plastic material like polyvinyl chloride (PVC). The Danakil depression is located in Afar, Ethiopia, Africa with coordinates of 14° 14′ 30.1″ (14.2417°) north (latitude) and 40° 18′ (40.3°) east (longitude). It is within the Great Rift Valley in northeastern Ethiopian, to the southeast of Eritrea and of west Djibouti. It covers about 5,000 km^2^, the center of which is known as the Dallol depression and is 120 m below sea level. It is one of the hottest and most inhospitable places on earth (Morel, 2005). Danakil has the highest average temperatures on Earth, exceeding 34°C every day of the year and reaching 55°C in the summer.

### Moisture and pH determination

The salt samples were ground using a mortar and pestle, and 5 g of the salt powder was placed in an oven at 105°C until it reached a constant weight, and then, it was allowed to cool. The moisture content was calculated based on the weight difference before and after baking. Ten grams of salt was mixed with 50 ml of distilled water (DW) in a ratio of 1:5, and the pH was measured after complete dissolution.

### DNA extraction

Microbial DNA extraction from salt samples was carried out using a slightly modified method as described in Henriet et al. (2014). Afdera salt was used as a model. The three basic extraction steps tested were (i) dissolution of stored salt sample with DW, (ii) cell concentration by centrifugation, and (iii) bead beating, chemical lysis, and DNA recovery. A 10 g sample of salt was dissolved to 50 ml of DW until complete dissolution. As the microbial content in commercial salt is very low, the cells with soils were concentrated by centrifugation (5 min, 10,000 × g and 4°C). Cell bead beating, chemical lysis, and DNA recovery were achieved with PowerSoil DNA isolation kit (MO-Bio Laboratories Inc., Carlsbad, CA, USA) following the manufacturer's instructions. A Quawell Q3000 UV spectrophotometer (San Jose, USA) was used to quantify the DNA.

### Polymerase chain reaction (PCR) and pyrosequencing

Standard methods were used to enumerate the approximate bacterial and archaeal loads in the four metagenomic samples. Purified DNA was amplified using primers targeting the V1–V3 regions of the prokaryotic 16S rRNA gene. The primers used for bacteria were V1-9F (5′-CCTATCCCCTGTGTGCCTTGGCAGTCTCAGACGAGTTTGATCMTGGCTCAG-3′); and V3-541R (5′-CCATCTCATCCCTGCGTGTCTCCGACTCAG*X*ACWTTACCGCGGCTGCT-GG-3′) (Chun et al., 2010). The primers used for archaeal organisms were AV1-21F (5′-CCTATCCCCTGTGTGCCTTGGCAGTCTCAGAGTCCGGTTGATCCYGCCGG-3′) and AV3-519R (5′-CCATCTCATCCCTGCGTGTCTCCGACTCAG*X*GAGGTDTTACCGCGGCK-GCTG-3′) (Hur et al., 2011). The *X* denotes a 7–11 nucleotide barcode that was unique for each subject, followed by a common linker for bacteria (AC) and archaea (AG), and underlining indicates the gene specific section. PCR amplification was performed in a 50 μl volume containing 0.5 μl of 5 U/μl Taq DNA polymerase, 5 μl of 10 × PCR reaction buffer, 4 μl of 0.2 mM dNTP mix, 2 μl of each primer, and 1 μl of template DNA with the following thermal cycler (PTC-200 DNA Engine; MJ Research, CA, USA) conditions: initial denaturation at 95°C for 5 min; 30 cycles of denaturation at 95°C for 30 s, annealing at 55°C for 30 s, and extension at 72°C for 60 s; with a final extension at 72°C for 7 min. The size and the homogeneity of PCR products were confirmed by gel electrophoresis. PCR products were purified using a QIAquick PCR Purification Kit (Qiagen, Hilden, Germany). Bands of less than 300 base pairs were removed using a QIAquick Gel Extraction Kit (Qiagen) in a subsequent gel electrophoresis. The DNA sequencing was performed by Chunlab, Inc. (Seoul, Korea) with a Roche/454 GS FLX Titanium platform (454 Life Sciences, Roche Branford, USA) according to the manufacturer's instructions.

### Sequence data processing and statistical analysis

The sequencing reads from the different samples were separated by unique barcodes. The sequences of the barcode, linker, and PCR primers were then removed from each side of the original sequencing reads. The resulting sequences were subjected to a filtering process where only reads containing 0 to 1 ambiguous base calls (Ns) and 300 or more base pairs were selected for the final bioinformatics analyses. Nonspecific PCR amplicons that showed no match with sequences in the 16S rRNA gene database by BLASTN search (expectation value of > e^−5^) were also removed from later analyses. Chimeric sequences were removed by analyzing the differences in BLASTN-based sequence similarity patterns between the first half and second half of a sequence. When the first and second halves were identified as sequences of different bacterial orders, the sequence was regarded as a chimera and eliminated (Edgar et al., 2011). The filtered reads of the 16S rRNA gene sequences were identified via BLAST search against the EzTaxon-e database (http://www.ezbiocloud.net/) (Chun et al., 2007), which contains 16S rRNA gene sequences of type strains with valid published names and representative species-level phylotypes for either cultured or uncultured entries in the GenBank public database, with complete hierarchical taxonomic classification from the phylum to the species level.

The pyrosequences from the samples were analyzed using CLcommunity 3.0 (Chunlab Inc., Seoul, Korea). Operational taxonomic units (OTUs) were defined with a 3% divergence threshold using the average neighbor-clustering algorithm. The cutoff value for assigning the sequence to a species-level phylotype was ≥97% similarity. The cutoff value for a genus-level cluster was ≥94% similarity. CD-HIT was used for massive clustering of amplified sequences (Fu et al., 2012), and corresponding graphical representations were generated using this program. Alpha diversity indices such as the Abundance-based Coverage Estimator (ACE), Chao1 estimator, Shannon diversity index, and Good's coverage estimator were calculated using Mothur software version 1.28.0 (Schloss et al., 2009) to estimate species richness. The accession numbers of the GenBank sequences with the highest similarity to the sequence cluster were used to assign a tentative genus, if the genus level sequence cluster was not identified as a valid bacterial or archaea genus. The overall phylogenetic distance between each pair of communities was estimated using the Fast UniFrac web interface (Hamady et al., 2010) and visualized by a principal coordinate analysis (PCoA). In addition, similarities with various metagenomic datasets (Asia and Europe seawater) were used to evaluate community composition. The seawater datasets are open resources provided by Chunlab Inc. and show microbial diversity of Asia and European seawater. Metagenomic sequences were deposited in MG-RAST (http://metagenomics.anl.gov/) under deposition numbers EAS (mgm4717086.3 & 4717097.3), ERS (mgm4717082.3 & 4717088), KJS (mgm4717099.3 & 4717080.3), and KTS (mgm4717084.3 & 4717095.3).

## Results

### Moisture, pH, and production process

The Ethiopian Afdera Lake salt (EAS) and rock salt (ERS) were sampled from Ethiopia in January 2014, and Korean Jangpan salt (KJS) and Topan salt (KTS) were sampled from Korea in February 2015. Earthen ponds, salt plains, mudflats, and synthetic materials were used for the production of EAS, ERS, KTS, and KJS, respectively. According to Table 1, KJS had the highest moisture content among the sampled salts, whereas the lowest was recorded for ERS. The pH range of salts was 7.56–8.20, which indicated that they were produced in alkaline environments.

**Table 1:** **General information and some physicochemical characteristics of the commercial salts**.

**Parameter/condition**	**EAS**	**ERS**	**KJS**	**KTS**
Moisture content	4.5%	1.9%	11.3%	6.3%
pH	8.07	7.56	8.20	7.88
Production source	Lake Afdera brine	Salt plain	Sea	Sea
Form	White crystal	Salt bar	White crystal	White crystal
Production condition	Earthen pond	Salt plain	Synthetic material	Mudflats
Major use	Food preparation, salting of beaf	Food preparation	Food preparation, salting of fish	Food preparation, salting of fish
Sample date	January 2014	January 2014	February 2015	February 2015
Origin place, country	Danakil depression, Ethiopia	Danakil depression, Ethiopia	Shinan, Korea	Shinan, Korea

### Quantitative characterization of microbial diversity

A total of 65,256 valid reads with an average read length of 414 base pairs were obtained (Table 2). The amplicons comprised 48.22–61.40% bacteria, 37.72–51.26% archaea, and 0.507–0.86% eukarya. A total of 0.005–0.009% of reads were considered unclassified. In prokaryotes, taxonomic classification and quantification of the amplicon sequencing data showed that the bacteria belonged to 24 described phyla and 7 unaffiliated phyla. The bacterial phyla *Proteobacteria, Bacteroidetes, Actinobacteria*, and *Firmicutes* were dominant. However, the proportion of each phylum varied greatly among the salts. For example, *Proteobacteria* were the most abundant, comprising 83.89, 46.55, and 46.54% in KJS, KTS, and EAS, respectively. The second most abundant phylum was *Bacteriodetes*, which represented a relatively high proportion of bacteria in EAS (39.86%), and smaller proportions in KTS (26.55%) and KJS (8.81%). The third most abundant phylum was *Actinobacteria*, which ranged in abundance from 27.07, 13.32, 2.06, and 1.79% in ERS, KTS, KJS, and EAS, respectively. In contrast, the *Firmicutes* represented a large percentage (27.22%) of the bacteria, which, in ERS, was slightly more abundant than the *Proteobacteria* (26.59%) and *Actinobacteriodes* (22.07%). Interestingly, ERS and EAS had unique phyla: *Streptophyta, Chloropflexi*, and *Acidobacteria*.

**Table 2:** **Number of sequences and OTUs, Shannon diversity and Choa indices, coverage and valid number of reads of archaeal and bacterial metagenomics from samples**.

**Organism**	**Clone library sample**	**Valid reads**	**No. of phylotypes or OTUs**	**Choa1**	**Shannon diversity index**	**Goods lib. coverage**
Archaea	EAS	6002	952	1835.750	5.248	0.916
	ERS	5589	432	749.203	4.034	0.964
	KJS	2770	350	684.467	4.673	0.937
	KTS	14931	1503	2391.570	5.485	0.956
Bacteria	EAS	11346	716	1281.841	3.594	0.966
	ERS	7788	2266	3439.151	7.008	0.867
	KJS	8821	631	1129.245	3.883	0.965
	KTS	8009	848	1392.448	4.935	0.949

**Figure 1:**
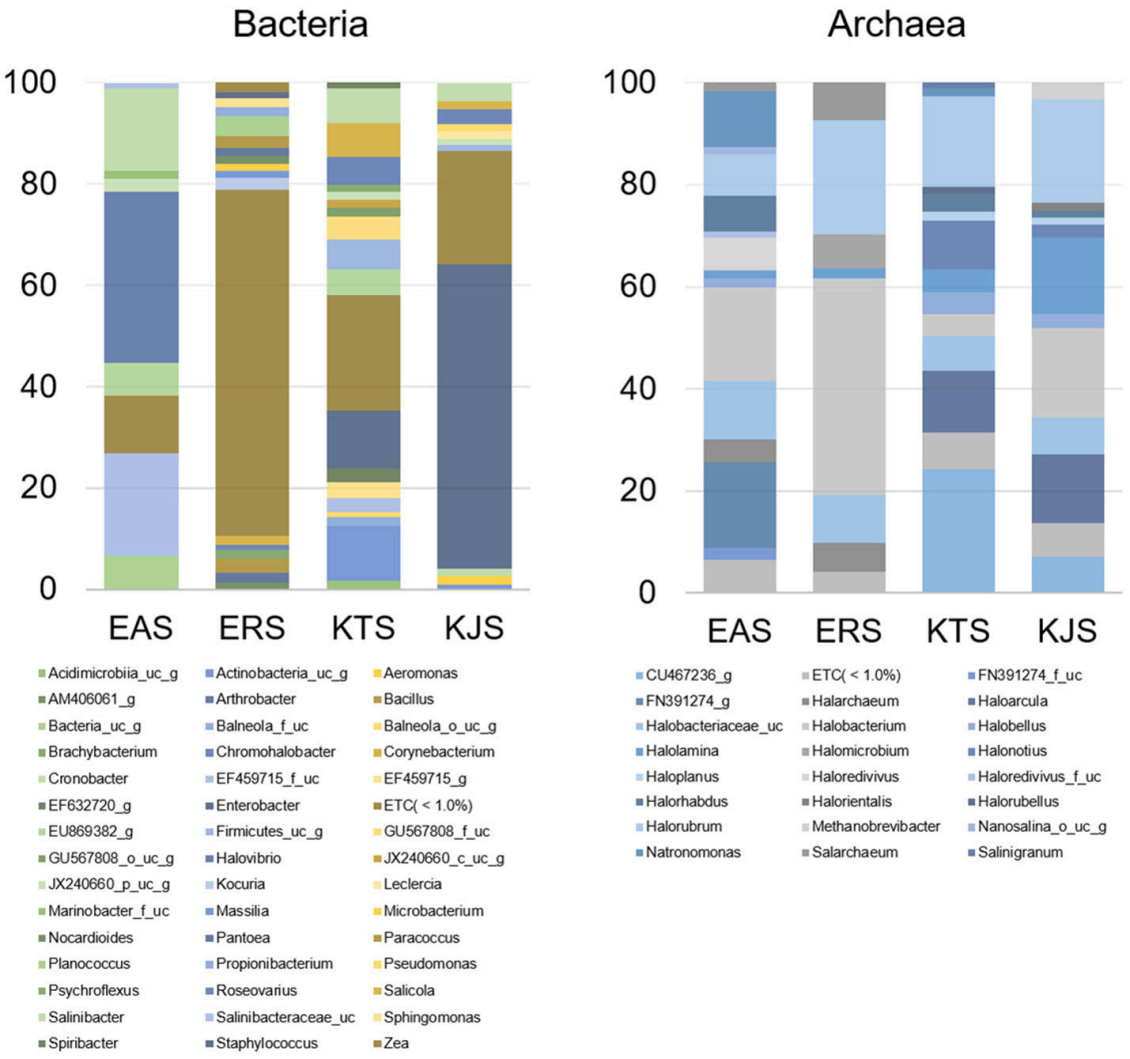
**Average composition of selected communities of metagenomically identified bacterial and archaeal genera of salt samples**.

Further classification at the class level indicated that the bacterial communities varied markedly among the salts. For instance, KTS and EAS were dominated by three classes: *Gammaproteobacteria, Deltaproteobacteria*, and the unaffiliated *Rhodothermus-c*. The majority of the reads belonged to the phyla *Proteobacteria* and *Bacteroidetes*, although *Gammaproteobacteria* were heavily represented in KJS (73.22%). In addition, unaffiliated *Actinobacteria_uc* (10.84%, phylum *Actinobacteria*) and *Sphingobacteria* (20.9%, phylum *Bacteriodetes*) were better represented in KTS and EAS, respectively. Intriguingly, the ERS bacterial community was dominated by unaffiliated *Actinobacteria*_c (25.23%), *Bacilli* (16.18%), and *Clostridia* (10.79%) in the phyla *Actinobacteria* and *Firmicutes. Alphaproteobacteria* in the phylum *Proteobacteria* were heavily represented in ERS (12.46%), KTS (10.21%), and KJS (8.03%). Unaffiliated *Bacteria_uc_c* (6.6%), *Betaproteobacteria* (4.55%), and unaffiliated *Rhodothermus_c* (4.02%) were most common in EAS, ERS, and KJS, respectively. The dominant genus in each sample is presented in Figure 1. Further comparison of the two salt groups (from the Danakil depression in Ethiopia and Shinan in Korea) showed the presence of many bacterial groups unique to each area (Table 3). For instance, *Planococcus, Arthrobacter, Brachybacterium, Micrococcus, Thermoleophilia*, and *Thermomicrobia* were occurred only to EAS or ERS, or were present in both salts. Similarly, *Salicola, Aeromonas, Spiribacter, Leclercia, Coscinodiscophyceae*, and *Chlorophyceae* were only present in KTS and KJS.

**Table 3:** **Some exclusively recorded bacteria genera identified in the Danakil depression (EAS and ERS) and Shinan (KJS and KTS) salts from the metagenomic dataset**.

**Genus name**	**EAS**	**ERS**	**KJS**	**KTS**	**Sum**
*Planococcus*	9	307	ND^*^	ND	316
*Arthrobacter*	8	140	ND	ND	148
*Brachybacterium*	1	144	ND	ND	145
Un affiliated AM406061_g	4	112	ND	ND	116
*Microbacterium*	4	97	ND	ND	101
*Micrococcus*	2	74	ND	ND	76
*Romboutsia*	9	57	ND	ND	66
Un affiliated EF602759_f_uc	5	60	ND	ND	65
*Devosia*	1	52	ND	ND	53
*Tessaracoccus*	2	50	ND	ND	52
*Janibacter*	1	46	ND	ND	47
*Ornithinimicrobium*	1	43	ND	ND	44
*Lactobacillus*	2	41	ND	ND	43
Un affiliated Bacillaceae_uc	3	40	ND	ND	43
*Dietzia*	2	41	ND	ND	43
*Salicola*	ND	ND	141	524	665
Un affiliated EF459715_g	ND	ND	64	254	318
Un affiliated EF632720_g	ND	ND	59	220	279
Un affiliated Acidimicrobiia_uc_g	ND	ND	46	139	185
*Aeromonas*	ND	ND	145	26	171
*Spiribacter*	ND	ND	84	83	167
*Leclercia*	ND	ND	144	17	161
*Psychroflexus*	ND	ND	37	118	155
*Cronobacter*	ND	ND	125	18	143
*Gramella*	ND	ND	20	68	88
Un affiliated Balneola_c_uc_g	ND	ND	31	39	70
Lutimaribacter	ND	ND	15	46	61
Un affiliated EU735698_g	ND	ND	17	37	54
*Dunaliella*	ND	ND	20	26	46
Un affiliated HE576995_g	ND	ND	29	17	46

ND* (not detected).

Within the domain Archaea, all salts were represented by only one archaeal phylum, *Euryarchaeota*. Of the overall archaeal community, 91.58% belonged to the class *Halobacteria*, while the remaining 7.58, 0.83, and 0.01% were members of the *Nanoarchaea, Methanobacteria*, and *Thermococci*, respectively. Further examination of the salt metagenomic data indicated that the family *Halobacteriaceae* dominated the archaeal communities, representing 100, 99.18, and 96.68% of archaea in KTS, ERS, and KJS, respectively, and a relatively low percentage (69.88%) of archaea in EAS. Different unaffiliated groups in class *Nanohaloarchaea* made up more than 30% of the archaea in EAS. In addition, *Methanobacteriaceae* (3.32%) was only present in KJS. At the genus level, the top five dominant archaeal genera were *Halorubrum* (20.25%), *Halobacterium* (17.47%), *Halolamina* (14.98%), *Haloarcula* (13.5%), and unaffiliated *Halobacteriaceae*_uc (7.22%) in KJS; unaffiliated CU467236-g (24.14%), *Halorubrum* (17.83%), *Haloarcula* (12.0%), *Halonotlus* (9.54%), and unaffiliated *Halobacteriaceae_uc* (6.91%) in KTS; *Halobacterium* (18.18%), unaffiliated FN391274_g (16.84%), unaffiliated *Halobacteriaceae*_uc (11.68%), *Natronomonas* (10.96%), and *Halorubrum* (8.3%) in EAS; and *Halobacterium* (42.39%), *Halorubrum* (22.49%), unaffiliated *Halobacteriaceae*_uc (9.43%), *Salachaeum* (7.32%), and *Halomicrobium* (6.51%) in ERS (Figure 1). In addition, *Salinigranum, Halorientalis*, unaffiliated *EF533953*_g, *Halomicroarcula*, and *Halosimplex* were some of the genera that were found only in the Shinan salts, whereas unaffiliated *FN391274*_g, *FN391274*_f_uc, *EU869371*_f_uc, and *Halorussus* were some of the archaeal genera that were only recorded in the Danakil depression salts (Table 4).

**Table 4:** **Some exclusively identified archaea genera from the Danakil depression (EAS and ERS) and Shinan (KTS and KJS) salts from the metagenomic data set**.

**Genus name**	**EAS**	**ERS**	**KJS**	**KTS**	**Sum**
*Salinigranum*	ND^*^	ND	22	168	190
*Halorientalis*	ND	ND	44	89	133
Un affiliated EF533953_g	ND	ND	9	81	90
*Halomicroarcula*	ND	ND	4	81	85
*Halosimplex*	ND	ND	9	59	68
Un affiliated AM947497_g	ND	ND	1	35	36
*Halogeometricum*	ND	ND	1	6	7
*Haladaptatus*	ND	ND	1	2	3
Un affiliated FN391274_g	1011	5	ND	ND	1016
Un affiliated FN391274_f_uc	145	25	ND	ND	170
Un affiliated EU869371_f_uc	57	10	ND	ND	67
*Halorussus*	2	28	ND	ND	30
Nanosalina_f_uc	14	5	ND	ND	19
Un affiliated EU869371_g	11	1	ND	ND	12

ND* (not detected).

**Figure 2:**
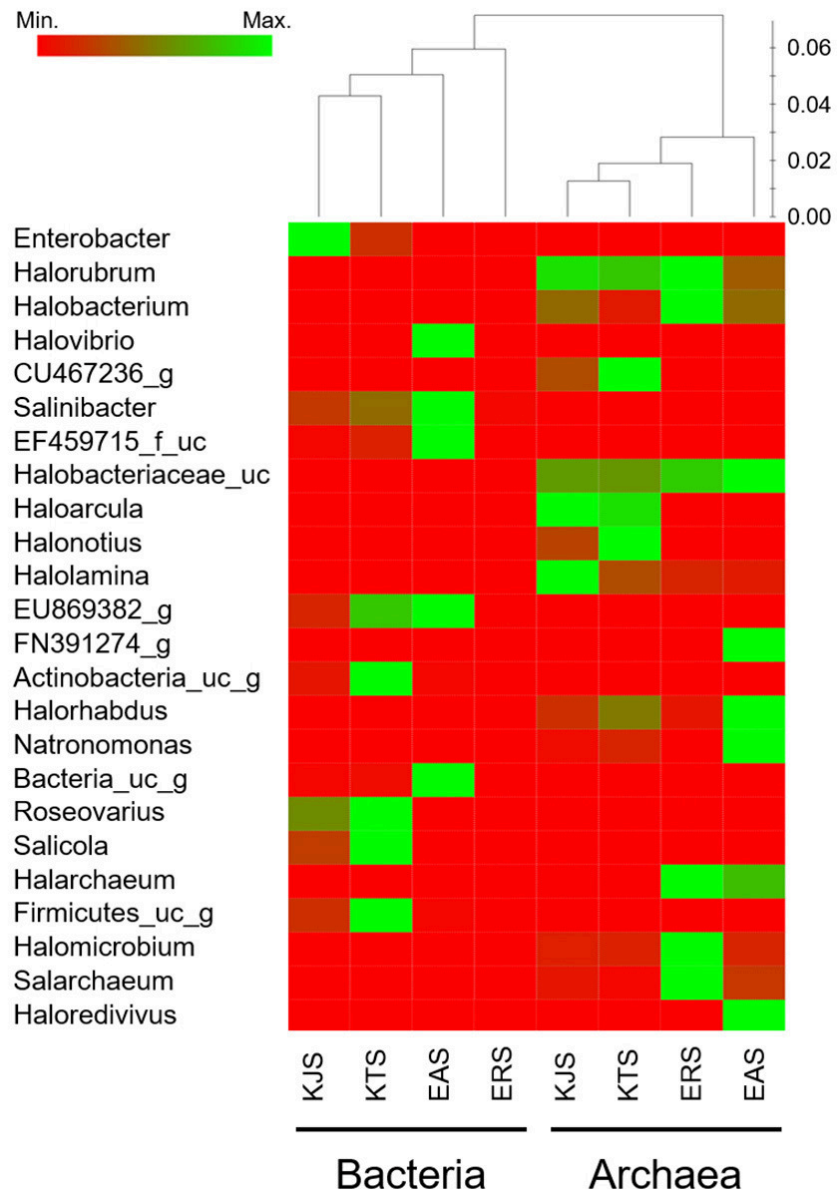
**Heat map showing the relative abundances and distribution of representative 16S rRNA gene tag sequences classified at the genus level**. The color code indicates the differences in the relative abundance from the mean, ranging from red (negative) through black (the mean) to the green (positive).

A heat map analysis of the bacterial and archaeal communities at the genus level revealed distinctions in diversity in the salts. For example, *Enterobacter*, the most abundant genus, was detected only in KJS and KTS, whereas *Salinibacter*, observed in KTS, KJS, and EAS, was more dominant in EAS than in the other two salts (Figure 2). In addition, more sequences associated with *Roseovarius, Salicola*, and unaffiliated *EU869382_g* were detected in KTS and EAS than in the other salts. Unaffiliated *Actinobacteria*_*uc_g* was mainly present in KTS, and unaffiliated *Bacteria*_uc_ and *Halovibro* were mainly present in EAS. Among the archaea, *Halorubrum, Halobacterium, Haloarcula, Halonotius, Natromonas, Halarchaeum, Halomicrobium*, and *Salarchaeum* were detected in each salt. *Halolamina, Halonotius, Halarchaeum*, and *Natronomonas* were among the genera that were well represented in KJS, KTS, ERS, and EAS respectively. *Haloredvivus* was well represented only in EAS.

### Diversity estimation and microbial community structure

**Figure 3:**
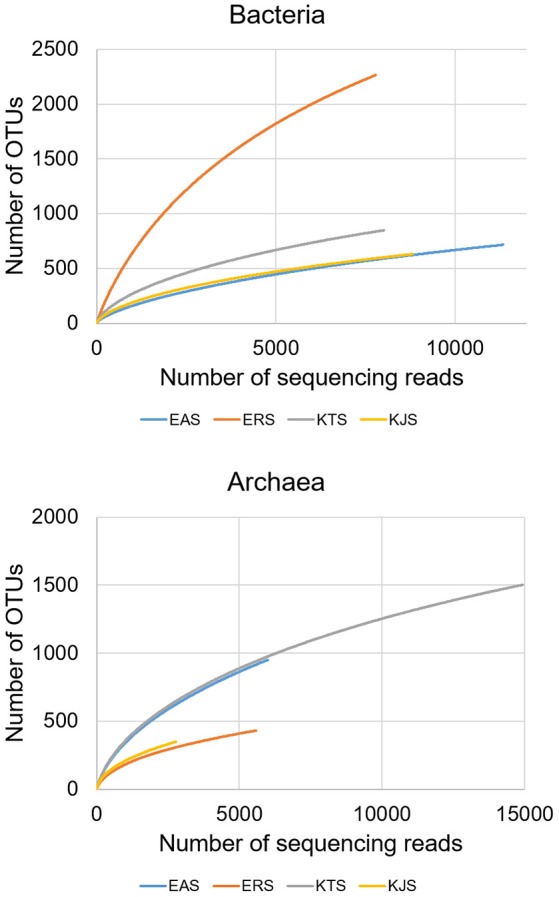
**The rarefaction curves of pyrosequenced bacteria (upper panel) and archaea (bottom panel) communities of the four salts**.

Bacterial and archaeal diversity were estimated using the Shannon index, as well as non-parametric richness (Chao1) and Good's Coverage estimations (Table 2). The alpha diversity (species richness) varied across samples. These results indicated that among the four salts, ERS had the highest bacterial diversity, followed by KTS and EAS (Shannon indices of 7.008, 4.935, and 3.883, respectively). KJS showed the lowest value for bacteria (Shannon index of 3.883). In terms of bacterial richness, ERS (3439.15) had the highest chao1 value, and KJS (1129.245) had the lowest. Similarly, archaeal richness was highest in KTS (2391.57) and lowest in KJS (684.467). Furthermore, rarefaction curves were computed for the four datasets based on the number of reads and species to evaluate the extent of diversity captured (Figure 3).

### Comparison within and other biomes

**Figure 4:**
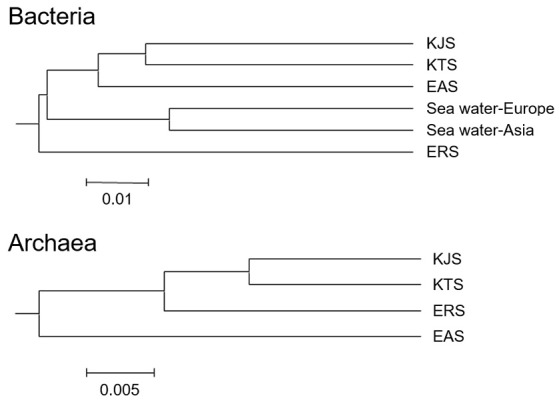
**Clustering analysis for the samples classified as archaea and bacteria using the UniFrac service after genome sequencing**. The scale bar indicates the distance between clusters in UniFrac units (data generated with CL community, Chun Lab Inc.).

**Figure 5:**
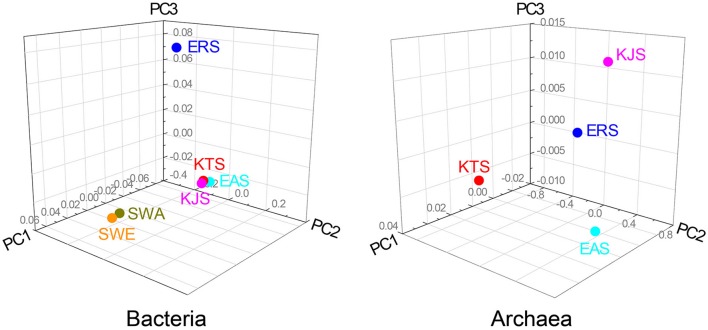
**UniFrac distance-based Jackknife clustering of bacteria and archaea communities associated with difference salts**. Asian seawater (SWA) and Europian seawater (SWE) samples as the control were also included in this analysis. Unifrac PCoA image were captured from 3D UniFrac PCoA to illustrate differences in the microbiota among the different salts. The following UniFrac analysis were based on the OTU data, with the first three principal coordinates (PCs) shown: unweighted UNiFrac with PC1 (66.71%, 35.71%), PC2 (0.00, 0.00), and PC3 (9.23, 9.23).

The constitutions of the 16S rRNA gene sequences in salt-based and in other similar environments, were assessed using the UniFrac clustering method. This statistical analysis showed that all of the archaeal communities from the four salt samples clustered together. Interestingly, the bacterial communities in the salts were significantly different from each other and even more different from other communities that were used for comparison; they were placed in different clusters as indicated by the PCoA based on the unweighted UniFrac distances (Figures 4, 5). The plot showed that the bacteria of KTS and KJS clustered into one group. This is because many of the gene categories showed similar abundance levels. However, EAS and ERS did not cluster with KTS and KJS. Furthermore, clustering with similar environmental gene sequences (Asian and European sea bacteria, data taken from the Chunlab database) showed separate clustering of the bacterial communities in the salts. Salinity and other environmental factors may be an important factor in determining the level of similarity between microbial communities. Finally, a taxon exclusive-OR (XOR) analysis showed the presence of many unique genera of archaea and bacteria in each sample. ERS had many unique genera, including *Pantoea, Erminia, Weissella, Microvirga*, and *Caryophanon*. In addition, *Oceanicella* and *Oceaniovalibus* in KTS, *Salmonella* and *Roseibacterium* in KJS, and *Dactococcospsis* and *Halorhodospira* in EAS were the genera that were only detected in one salt.

## Discussion

In this study, we performed amplicon sequencing analyses to survey the microbial content of four commercial salts that were produced by solar evaporation of hypersaline waters and halite mining (Table 1). It is a well-known property of many halophiles to be polyploid and also to harbor multiple unrelated 16S rRNA gene copies in one cell (Oren, 2012). Our previous studies on haloarchaeal taxonomy also revealed that the haloarchaeal taxa such as *Halostella salina* (Song et al., 2016), *Halapricum salinum* (Song et al., 2014), and *Halorubrum halophilum* (Yim et al., 2014), have at least two different copies of the 16S rRNA gene. Because it is accurate to consider all of the reported heterogeneous 16S rRNA gene sequences for the phylogenetic study of the haloarchaea, we used the EzTaxon-e database containing the unrelated 16S rRNA gene copies of the haloarchaea to analyze haloarchaeal community profiles in the commercial salt samples.

Salted, fermented foods and extreme environments have been reported to maintain a high density of halophilic bacteria and archaea dominated primarily by *Proteobacteria, Bacteroidetes, Actinobacteria, Firmicutes*, and *Euryacheota* (Keshri et al., 2013; Fernandez et al., 2014). In this study, the microbial diversity of the salts revealed that the bacteria phyla *Proteobacteria, Bacteroidetes, Actinobacteria*, and *Firmicutes* were dominant in at least one of the salts, whereas the archaeal phylum *Euryacheota* was dominant in all salts. Most of extreme halophilic archaea are classified in class Halobacteria under one phylum Euryarchaeota while halophilic bacteria are distributed across several phyla.

In the amplicon sequencing data, we detected 16S rRNA gene sequences related to members of the genera *Enterobacter, Halovibrio, Roseovarius, Salicola*, and *Salinibacter* in one or more than one of the salts (Figure 1). Most of these genera are common in salted foods and hypersaline environments (Kim et al., 2012; Fernandez et al., 2014; Pandit et al., 2015). Remarkably, we observed the genus *Enterobacter* only in Shinan (KTS and KJS); this genus is not common in salted and fermented foods. In some salt samples, the study recorded genus like *Salmonella* and *Entrobacter* which are common in domestic wastes (Vrints et al., 2007) needs further study whether they are contaminated from human wastes. Interestingly *Enterobacter* is much more abundant in KJS than other salts. In order to determine whether the KJS production method is related to Enterobacter flourishing, further study is also needed. On the other hand this genus was almost absent from Danakil depression salts (EAS and ERS), perhaps because the Danakil depression is rather hot for *Enterobacter* survival, as their optimal growth temperature is 30°C (Grimont and Grimont, 2006). This idea is supported by the finding of *Arthrobacter, Choracidobacterium, Thermoleophilia, Thermomicrobia*, and *Planococcus* bacteria in EAS and ERS, which were recorded in hot environments (Pearson and Noller, 2011; Ghati et al., 2013; Stan-Lotter and Fendrihan, 2015). In addition, *Brachybacterium, Romboutsia*, and many other bacterial genera were only detected in the Danakil depression salts. Similarly, *Salicola, Aeomonas, Spiribacter, Leclercia*, and *Psychroflexus* were detected only in Shinan salts (Table 3). It is important to note that the presence of many unique bacterial genera in the salts of the Danakil Depression and Shinan is a result of local and regional environmental factors that influence the assembly of bacterial communities (Lindstrom and Langenheder, 2012).

Within the domain Archaea, the genera *Halorubrum, Halobacterium, Haloarcula, Halonotius, Natromonas, Halarchaeum, Halomicrobium*, and *Salarchaeum* were recorded in all salts. *Halorubrum, Halobacterium*, and *Haloarcula* also dominated the archaeal communities in different salted seafoods and kimchi (Chang et al., 2008; Roh et al., 2010). In addition, at least one of the archaeal genera has been found to dominate a hypersaline environment (Ochsenreiter et al., 2002; Youssef et al., 2014; Oren, 2015). In contrast, *Haloquadratum*, which is one of the dominant archaeal genera in salterns (Oh et al., 2010; Dillon et al., 2013), appeared to be a minor member in commercial salts. Despite the geographical separation, EAS from the Danakil depression and KTS from the Shinan share a very similar community structure, as most of the dominant bacterial and archaeal groups were the same. This might be attributed to the production of salts in earthen ponds and mudflats, where salt may be contaminated with soil during the production processes, because most of these bacterial groups have also been detected in saline sediments (Lopez-Lopez et al., 2010; Kim et al., 2012). Comparison of the rarefaction curves with the number of OTUs determined by the Chao1 richness estimator and other diversity indices revealed that the two geographical areas had high microbial diversity, likely because of the intrinsic features of these ecosystems that provide specialized niches for the evolution of unique microbial communities (Smith et al., 2012). A comparison of the salt datasets with those from Asian and European seawater resulted in different clusters in the PCoA plot (Figures 4, 5). The overall microbial community structure of all salts in the current study was similar to that of the Asian and European seawater which were used to evaluate community comparison. This similarity in salt metagenomics is based on similar proportions of Gammaproteobacteria, Deltaproteobacteria, Actinobacteria_uc, and Betaproteobacteria in the bacteria communities. Archaeal communities were also similar, as *Halorubrum, Halobacterium, Haloarcula, Halonotius, Natromonas, Halarchaeum, Halomicrobium*, and *Salarchaeum* were detected in each salt.

In conclusion, the exploration of microbial diversity in commercial salts revealed that they contained various bacteria and archaeal taxa. The salts contained surprisingly diverse microbial communities. The bacteria were dominated by the phyla *Proteobacteria, Bacteroidetes, Actinobacteria*, and *Firmicutes*. While a substantial amount of the bacteria are predicted to be halophilic, some halotolerant strains producing exopolysaccharides may be able to survive on the crystal salts. The archaea, however, were represented only by Euryacheota. Unique genera were recorded in every salt sample; these merit further study. Recent analyses of the microbiota of patients who were the sources of nosocomial infections such as urinary tract infections, bacteremia, bowel diseases, and human colon diseases showed highly diverse bacteria and archaea from the *Enterobacteriaceae, Halobacteriaceae*, and *Methanobacteriaceae*, including close relatives of Enterobacter, Halobacterium, Halorubrum, and Methanobrevibacter (Grimont and Grimont, 2006; Samuel et al., 2007; Oxley et al., 2010). In addition, the genus *Romboutsia*, a spore-forming obligatory anaerobic bacterium, was isolated from the gastro-intestinal tract of a rat (Gerritsen et al., 2014). These disease causing genera were detected in this study. The first halophile which had been suspected of being pathogenic for man was *Pseudomonas enteritis* TAKIKAWA. It was isolated from a case of food-poisoning in 1955 (Okudaira et al., 1962). Recently, Bang and Schmitz (2015) reviewed the current knowledge of human mucosa-associated archaeal species, their interaction with the human immune system and their potential contribution to humans' health and disease. However, no concerted efforts have been undertaken to implicate archaea in human diseases. The full spectrum of outcomes from these archaea-human interactions, whether it includes tissue damage, or clinical disease, altered host physiology, remains a mystery. Effects on the health and immune response of haloarchaea are being investigated. Therefore, halophilic bacteria and haloarchaea ingested with salty foods and their roles in human health should be further investigated.

## Author contributions

AG, KP, and MW prepared metagenomic DNA from commertial salts and carried out other experiments. AG and JS(Shin) collected the commercial salts and characterized the physicochemical properties of the salts. AG, DL, JS(Sohn), MS, and SR analyzed the sequencing data and phylogenetical analysis. DL, SR, SL, and HL conceived the study, participated in its design and interpreted the data. HL coordinated the authors and wrote the manuscript with approvals of all authors. All authors are participated in editing of the manuscript.

### Conflict of interest statement

The authors declare that the research was conducted in the absence of any commercial or financial relationships that could be construed as a potential conflict of interest.

## Abbreviations

EAS: Ethiopian Afdera salt
ERS: Ethiopian rock salt
KJS: Korean Jangpan salt
KTS: Korean Topan salt
PCoA: principal coordinate analysis
OUT: operational taxonomic unit
XOR: exclusive-OR
SWA: seawater in Asia
SWE: seawater in Europe.
